# International research priority setting exercises in stroke: A systematic review

**DOI:** 10.1177/17474930221096935

**Published:** 2022-05-12

**Authors:** Stephanie Leitch, Monica Logan, Lucy Beishon, Terence J Quinn

**Affiliations:** 1 Institute of Cardiovascular and Medical Sciences, University of Glasgow, Glasgow, UK; 2 Department of Cardiovascular Sciences, University of Leicester, Leicester, UK

**Keywords:** Priority setting, research methods, stroke remove, systematic review, research prioritization, methodology

## Abstract

**Background:**

Agreeing on priority topics for stroke research can help make best use of limited funding, people, and time. Formal priority-setting exercises collate stakeholders’ opinions to reach consensus on the most important research questions. Several stroke research priority setting exercises have been published. Exploring commonalities and differences between these exercises could bring a better understanding of priority research topics.

**Aim:**

We collated and compared published stroke research priority setting exercises across international healthcare systems.

**Summary of review:**

Multidisciplinary, electronic literature databases were searched from 2000 to 2021, using a validated search syntax. Inclusion criteria were: full article; stroke focus (any subtype); prioritization method described; and lists priorities for research. Priorities were extracted, coded, and assigned to categories using thematic analysis. The Nine Common Themes of Good Practice and the Reporting guideline for priority setting of health research checklists were used to assess methodological and reporting quality respectively. From 623 titles assessed, 14 studies were eligible for inclusion, including 2410 participants and describing 165 priorities. The majority of priority setting exercises were conducted in high-income countries (86%, n = 12 articles), published between 2011 and 2021 (64%, n = 9), and included views of healthcare professionals (57%, n = 8), and stroke survivors (50%, n = 7). Caregivers (n = 3, 21%) were under-represented. The James Lind Alliance priority setting method was most commonly used (50%, n = 7). Priorities were grouped into 10 thematic categories. Rehabilitation and follow-up was the most common priority theme (15%, n = 25 priorities), followed by psychological recovery (14%, n = 23), pathology (14%, n = 23), and caregivers and support (14%, n = 23). Priorities differed by year and case-mix (stakeholder group and demographics) of respondents. No article was judged high quality for all aspects of method or reporting. Common limitations were around inclusiveness and evaluation of the exercise.

**Conclusion:**

Stroke research priorities are dynamic and context-specific. However, there was a common theme of prioritizing research that considered life after stroke. Future priority settings should consider the inclusion of nonindustrialized countries and stroke survivors with a range of impairments.

## Introduction

Stroke care strives to be evidence-based. Research has transformed stroke care,^1^ and through research, we can continue to meet contemporary stroke challenges. There is an urgent need to promote and support the global research agenda for stroke, particularly among low- and middle-income countries.^2^ Despite this, stroke remains a relatively underfunded research area, receiving five times less funding than cancer research.^3^ Thus, stroke researchers, funders, and policymakers face a fundamental problem, and there are many important questions but only limited resource to support the necessary research to answer them.

Priority setting seeks to identify key unanswered research questions by consulting a range of stakeholders with differing professional expertise (e.g. clinicians, researchers), or firsthand experience (e.g. patients and caregivers) of a condition of interest.^4^ Research prioritization provides a means of targeting resources to the areas of greatest perceived need.^4^ Priority setting exercises are impactful and are increasingly used to direct the international research and funding agenda.

A range of methods are available for priority setting, and there is no consensus on the optimal approach. The three most commonly used methods are those described by the Child Health and Nutrition Research Initiative (CHNRI), James Lind Alliance (JLA), or Delphi-based consensus.^4,5^ Each of these methods shares common features of collating views across stakeholders and systematically ranking priorities to produce an ordered list^4^ (Table 1).

**Table 1.** Description of three common prioritization methods.

Method	Background of method	Scope of approach	Methodological guidance available	Patient and public involvement
Child Health and Nutrition Research Initiative (CHNRI)	CHNRI was developed by the Global Forum for Health Research in Geneva, Switzerland to assist priority setting in health research investments	A systematic flexible method for prioritization. Stakeholders weigh priorities against predefined criteria and set thresholds for a minimum acceptable score that would be required for an idea to be considered a research priority	Published guidelines and a framework are available^6^	Stakeholders are involved in the process, but this varies depending on the nature of the research being prioritized, and is decided on a project-by-project basis
Delphi method	Delphi is a decision-making process developed to agree consensus on best practice, which has been adapted for use in research prioritization	A process used to arrive at group decision through several rounds of questionnaires completed by experts with responses aggregated and shared after each round	The Delphi method has been adapted for use in research prioritization but guidelines are available for the conduct of Delphi studies^7^	Stakeholders are involved in the process, but this varies depending on the nature of the research being prioritized, and is decided on a project-by-project basis
James Lind Alliance (JLA)	JLA was established specifically to involve patients and carers in identifying research priorities	A combination of surveys and workshop interactions between patients, carers, and healthcare professionals to identify and agree on a “top ten” list of research questions	JLA handbook provides methods guidance across all stages of the process	PPI is central to JLA methods, with patients and public represented on steering groups, and attempts to ensure representation in terms of numbers of responses to surveys and participants at meetings

Several stroke prioritization exercises have been published over the last decade, including a recent high-profile exercise led by the UK Stroke Association^8^ and JLA. However, priorities are likely to be contextual, and may vary according to the healthcare system, priority setting method employed, and the participants included.^9^ Comparing priority-setting exercises may highlight common themes in research questions and also allows us to explore the reasons for any differences.

The aim of this review was to provide a comprehensive overview of published stroke research priority exercises by collating and comparing published priority-setting exercises across international healthcare systems. As a secondary aim, we described the potential effect of healthcare setting, year of prioritization exercise, prioritization method, and stakeholder involvement on the resulting research priorities identified.

## Methods

This review followed best practices in evidence synthesis and was reported (where appropriate) according to PRISMA guidelines. All aspects of title searching, data extraction, synthesis, and quality assessment were performed by two trained reviewers (SL and ML) working independently and comparing results. Differences were resolved through consensus, with recourse to a third reviewer (TQ) for the final decision as necessary. Processes and data extraction proformas were piloted using two exemplar stroke research prioritization articles^10,11^ and refined as necessary. As a nonclinical synthesis, the protocol for this review was not eligible for registration on the PROSPERO resource.

### Search strategy and selection criteria

Multidisciplinary, electronic literature databases were searched from January 2000 to December 2021 inclusive. Stroke management has changed considerably since 2000, and we considered that studies prior to this year were unlikely to be relevant. Included databases were: Medline (Ovid), Embase (Ovid), Health and Psychosocial Instruments (Ovid), PsychINFO (EBSCO), and CINAHL (EBSCO). Search terms were based on concepts of stroke and prioritization, and both used validated syntax (Supplemental Material). Reference lists of included studies were searched, in addition to searching websites of key organizations involved in research prioritization or stroke research: James Lind Alliance, American Stroke Association, European Stroke Organization, Stroke Association, World Stroke Organization, and World Health Organization.

Inclusion criteria were: full published article (or in press); stroke focus (could include any stroke subtype); describes prioritization methods; and reports a list of research priorities. We set no limits on country or language. Following an initial screening, suitable studies were reviewed as abstract and, if relevant, full text.

### Data extraction and analysis

Stroke priorities from included studies were extracted and aggregated verbatim to create a summative long list of identified priorities. Individual research priorities were combined and categorized using a thematic analysis approach. Categories were created from common topics or themes. Each priority was assigned a category, or a new category was created. Categories were combined where possible. The process was iterative and continued until all priorities were categorized. We described the total number and percentage of priorities identified for each theme. Percentage agreement was used to determine the categorization agreement between researchers.

We planned to compare prioritization results according to healthcare setting (high- vs. low- and middle-income countries, based on World Bank definitions); year of priority setting (taken as year of publication if not given in the text); method of prioritization (CHNRI, JLA, Delphi, etc.); and stakeholder involvement (proportion of respondents who were stroke survivors, caregivers, researchers and clinicians). Where studies recruited stroke-survivors we noted any demographic or clinical detail given.

### Quality assessment

Quality assessment included two complementary approaches that allowed for assessment of method and reporting. To assess the prioritization method, we used the Nine Common Themes of Good Practice (9CTGP).^12^ This checklist covers domains of context, inclusiveness, information gathering, criteria, methods, comprehensive approach, transparency, and evaluation.^12^ Reporting of the prioritization exercise was assessed using REporting guideline for PRIority SEtting of health research (REPRISE) materials.^4^ The REPRISE checklist consists of 31 items over 10 domains: context and scope, governance and team, framework, stakeholders (participants), identification and collection of research priorities, prioritization of research topics/questions, output, evaluation and feedback, implementation, funding, and conflict of interest.^4^ Assessment did not use a threshold score, rather articles were assessed as an overall high standard, low standard, uncertain standard, or not reported. Reliability was determined by percentage agreement. Poorly conducted or reported studies were not excluded from the synthesis, rather the exercise was used to identify common areas of good and poor practice in priority settings. We present the number of items and percentage within each category of the checklist that were considered high quality.

## Results

**Figure 1.**
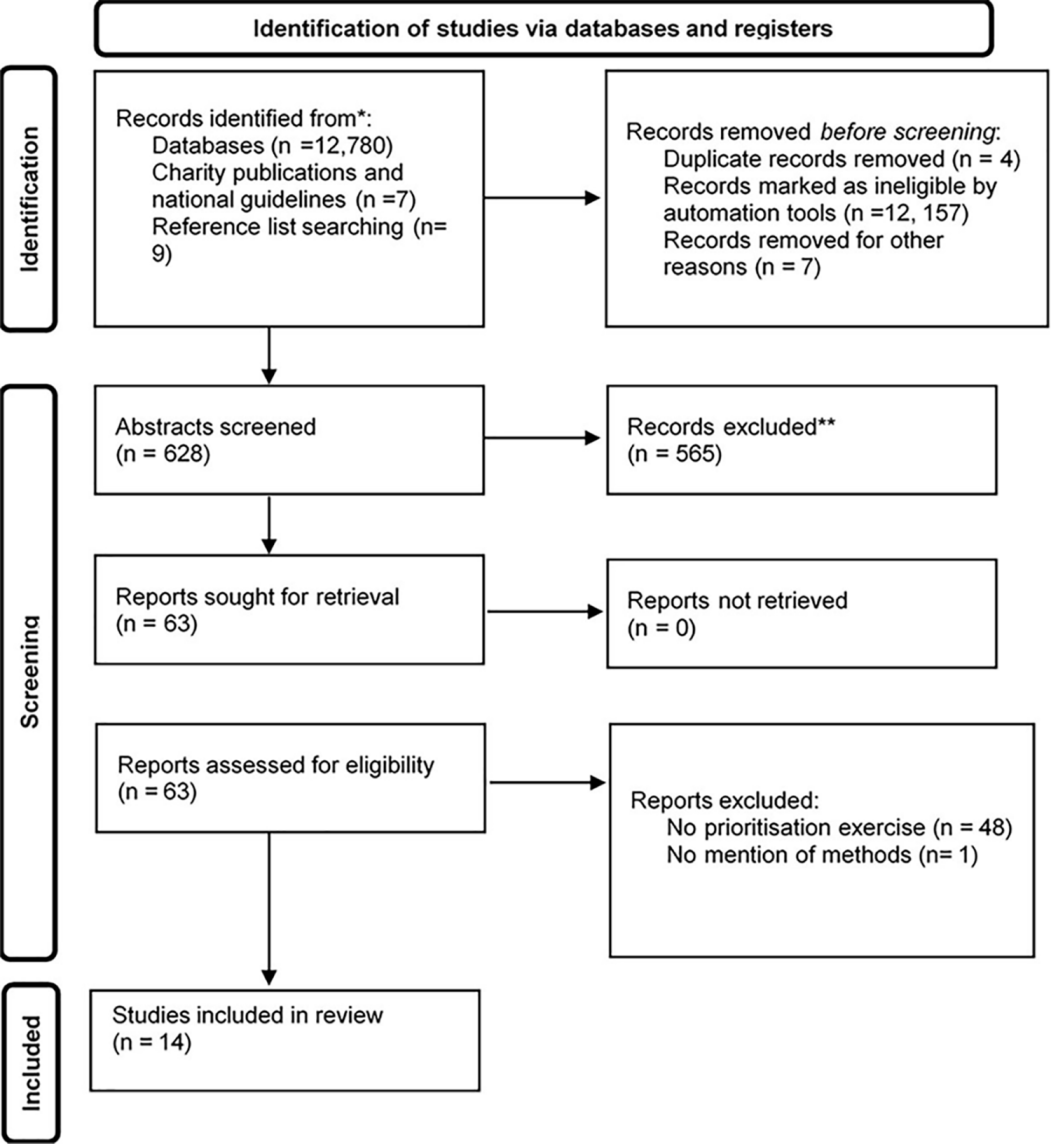
PRISMA diagram of systematic search.

In total, 12,796 studies were title screened, and 63 full-text articles were reviewed. Of these, 10 studies were eligible, with a further four studies identified from other sources. The 14 included studies sought an opinion from 2410 participants and described 165 priorities in total. One priority setting exercise, conducted by a stroke organization, was excluded as there was no description of the underlying methods (Figure 1).

### Summary of studies

Prioritization was predominantly conducted in high-income countries^8,10,11,13–21^ (86%, n = 12) with two studies^22,23^ assessing International opinion (Worldwide/Europe) and no priority setting specific to low- and middle-income settings (Tables 2 and 3). Over half of the prioritization exercises^8,10,13,15,17,19–22^ were published between 2011 and 2021 (64%, n = 9). There was heterogeneity in method, with (50%, n = 7) based on the JLA method or similar, that is, a process that began with questionnaires and was completed with consensus meetings.^10,13,15,16,19,20^ Three articles used a method that did not fit with any of the three traditional approaches to prioritization.^11,18,23^ Some of the included prioritization exercises had a specific theme of focus, for example, two articles majored on rehabilitation and two on the life after stroke.

**Table 2.** Descriptions of stroke research prioritization articles identified.

Author	Location	Number of participants	Leadership	Stakeholder groups	Methods	Focus on the prioritization exercise	Research priorities identified (n)
Alexandrov^11^	USA	–	National Institute of Neurological Disorders and Stroke Stroke Progress Review Group	“Experts” (researchers and clinicians)	Written report submission	Stroke-related basic and clinical science areas	6
Bayley et al.^14^	Canada	24	Canadian Stroke Network and Stroke Canada Optimization of Rehabilitation through Evidence (SCORE) team	Researchers, clinicians, lay stakeholders, stroke survivor	Modified Delphi approach	Stroke rehabilitation and research	8 (5 research and 3 knowledge translation)
Franklin et al.^19^	UK	46—Interim prioritization 22—Final Consensus	Academic	People with stroke aphasia, carers, and speech and language therapists	I. Survey II. Consensus meeting	Aphasia	10
Lannin et al.^17^	Australia	Round 1—14 Round 2—38 Round 3—56	Academic	“Experts” (researchers and clinicians)	Delphi approach	Research priorities of clinicians working in stroke rehabilitation	18
Meairs et al.^23^	Europe	–	European Commission	“Experts” researchers and clinicians)	Workshop	Stroke prevention, treatment and recovery	26
National Stroke Foundation^21^	Australia	–	National Stroke Foundation	National Stroke Foundation staff members, Research Advisory Committee	I. Environmental scan II. Priorities weighed against set criteria	Priorities for research funding	6
Pollock et al.^10^	UK	97—Interim prioritization 28—Final consensus	James Lind Alliance	Stroke survivors, carers, charities, researchers and clinicians	James Lind Alliance	Life after stroke	10
Rowat et al.^18^	UK	40	Scottish Stroke Nurses Forum	Nurses	Qualitative study of focus groups	Stroke nurses’ clinical research priorities	10
Rowat et al.^15^	UK	97—Interim prioritization 28—Final consensus	Scottish Stroke Nurses Forum	Nurses	James Lind Alliance	Stroke nursing	10
Rudberg et al.^20^	Sweden	589	Academic	Participants in efficacy of fluoxetine-a randomized controlled trial in stroke trial	Survey	Life after stroke	11
Sacco et al.^22^	Global	25	The World Stroke Organization Stroke Research Committee	World Stroke Organization board members (researchers and clinicians)	Survey	Stroke treatment, prevention, and recovery	6
Sangvatanakul et al.^16^	Australia	18	Academic	Members of the Working Age Group Stroke, stroke survivors, stroke carers	I. Survey II. Consensus meeting	Priorities for guideline recommendations with a low level of evidence (National Stroke Foundation Australia)	13
Stroke Association^8^	UK	Stage 2—1407 Stage 4—1154	Charity (Stroke Association) and National Institute Health Research (NIHR) and James Lind Alliance	Stroke survivors, healthcare professionals, stroke charities, researchers, carers	James Lind Alliance	Prevention, prehospital and acute care Rehabilitation and long-term care	20
Turner et al.^13^	UK	11	Academic	Stroke survivors, stroke charities, researchers and clinicians	I. Survey II. Interim prioritization meeting III. Consensus meeting	TIA and minor stroke	11

**Table 3.** The number and percentage of studies by publication date, geographical location, stakeholder group, and method utilized.

Demographic	Subcategory	n (%)
Publication dates	2000–2005	1 (7)
	2006–2010	4 (29)
	2011–2015	5 (36)
	2016–2021	4 (29)
Location	United Kingdom	6 (43)
	United States	1 (7)
	Canada	1 (7)
	Australia	3 (22)
	Sweden	1 (7)
	Europe	1 (7)
	Worldwide	1 (7)
Stakeholder group	Stroke survivors	6 (43)
	Researchers/scientific experts	6 (43)
	Stroke charities/organizations	7 (50)
	Healthcare professionals	8 (57)
	Stroke carers	3 (21)
Method	James Lind Alliance method or similar	7 (50)
	Focus group/workshop	2 (14)
	Delphi	2 (14)
	Single survey	2 (14)
	Panel consensus	1 (7)
	Written report submission	1 (7)

Studies involved a variety of stakeholders, with healthcare professionals being the most commonly consulted^8,10,13–15,17–19^ (57%, n = 8), followed by stroke survivors (50%, n = 7). Stroke caregivers were the least represented^8,16,19^ (21%, n = 3). We found no examples where a prioritization exercise was updated or repeated. Although there were various prioritization exercises from the UK that used the JLA approach, these were distinct with differing remits, stakeholders, and support.

Of the studies that included stroke survivors’ views, only four provided participant demographics.^13,16,19,20^ Three studies reported the age of stroke survivors (mean range: 56–86 years).^13,16,20^ In all four studies, the majority of participants had mild-to-moderate stroke severity (range: 70–100%),^13,16,19^ The median National Institute of Health Stroke Scale was 3,^20^ and one study was restricted to patients with aphasia.^19^ One study recruited working-age participants only.^16^ One study reported cognitive function of stroke-survivor participants with predominantly mild deficits reported (median Montreal Cognitive Examination Score: 24).^20^

### Prioritization themes

**Figure 2.**
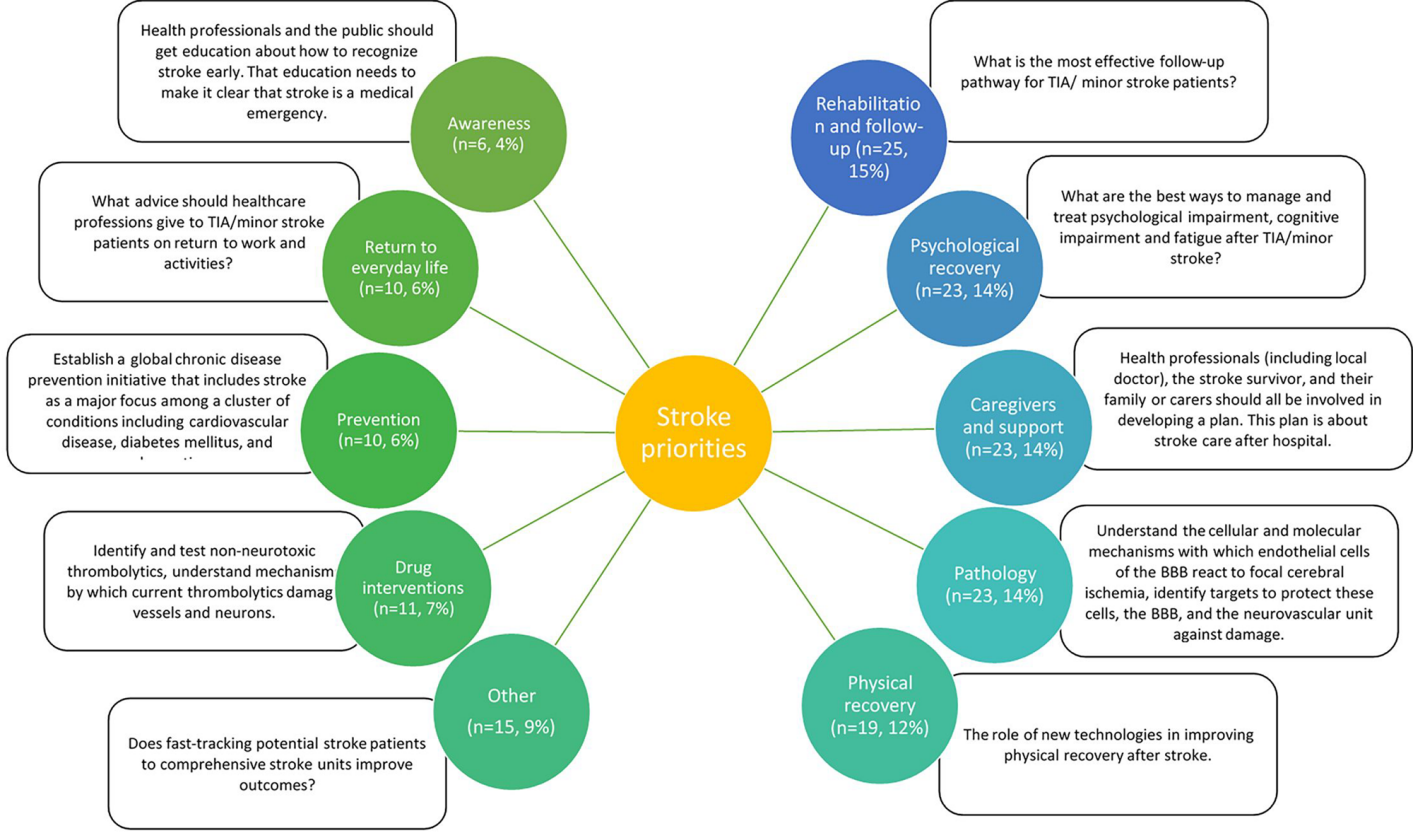
The frequency of prioritization categories mentioned within articles by number and percentage, and example research questions under each category. Priorities on the left ranked higher than those on the right in studies included in this review.

A total of 165 individual priorities were identified across included studies. Priorities were categorized into 10 themes, although theme 10 was “other” and included a mix of topics. The definition of each research theme and example research questions are provided in Supplemental Table 1. Rehabilitation and follow-up was the most common theme for research prioritization (n = 25, 15%). Of the top five themes, three had a direct link to longer-term issues following acute stroke (physical recovery, psychological recovery, rehabilitation, and follow-up), accounting for 41% of all priorities included. Figure 2 summarizes the frequency of priorities identified under each of the 10 themes. There was a high level of agreement (87%) between the two researchers in priority setting categorization.

We could not describe potential differences by healthcare setting or geography as no priority setting exercises from low- or middle-income countries were available. Differences were identified between the priorities identified in older (2000–2010) and more contemporary articles (2011–2021). Older priorities focused on pathology (33%, n = 21), while newer priority setting focused on rehabilitation and recovery (21%, n = 21) and psychological recovery (22%, n = 22). There has been a clear shift over time to include stroke survivors in these exercises as no articles predating 2010 included people with lived experience of stroke. When comparing stakeholder groups, those involving stroke survivors focused on rehabilitation and recovery (15%, n = 12), whereas those involving researchers focused on pathology (24%, n = 21). In general, healthcare professionals tended to have similar priorities to stroke survivors with a common focus on physical and psychological recovery. We summarized and divided priorities according to their stakeholder rating, and provide example research questions that could address key themes (Figure 2).

Further differences were demonstrated by the method of prioritization used. Those utilizing the JLA method were more likely to focus on caregivers (24%, n = 18) and aspects of psychological recovery (24%, n = 18). Delphi articles focused on recovery and rehabilitation (42% n = 11), while single survey articles focused on physical impairment (24%, n = 4). Studies relying on focus groups or workshops centered on pathology (44%, n = 16).

### Quality assessment

There was excellent agreement (87% and 97%) between reviewers on the 9CTGP and REPRISE checklists, respectively. Areas that were consistently well conducted across studies included information gathering (n = 13 items, 93% low risk), methods for deciding on priorities (n = 11 items, 79%), and transparency (n = 11, 79%). However, inclusiveness (involving a wide range of stakeholders) was only well conducted in five (36%) studies. In terms of reporting quality, descriptions of governance (n = 11 items, 26% reported well) and conflicts of interest (n = 12 items, 29% reported well) were poor. Some findings were common between both checklists, with context (n = 13 items, 93%, vs. n = 82 items, 80%) performing well on both tools. While implementation (n = 2 items, 21%, n = 2 vs. n = 8 items, 29%) and evaluation (14%, n = 1 items vs. 7%, n = 4 items) performed poorly in both (Table 4 and Supplemental Table 2).

**Table 4.** Results of nine common themes of good practice checklist.

	Turner	Bayley	Alexandrov	Sacco	Sangvatanakul	Lannin	Meairs	Rowat	Franklin	Rowat and Pollock	Pollock	National Stroke Foundation	Rudberg	James Lind Alliance
1. Context	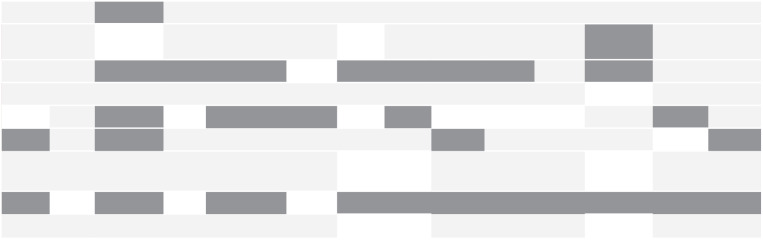													
2. Use of a comprehensive approach														
3. Inclusiveness														
4. Information gathering														
5. Planning for implementation														
6. Criteria														
7. Methods for deciding on priorities														
8. Evaluation														
9. Transparency														

Dark gray: not conducted/conducted poorly; white: uncertainty; light gray: well conducted.

## Discussion

We identified 14 stroke priority-setting exercises primarily conducted in high-income countries and using a range of methods and stakeholders. There was a move toward greater stroke-survivor involvement over time, with, in turn, a greater emphasis on the life after stroke in the topics prioritized. Most of the published priority-setting exercises were conducted in the last decade, suggesting that prioritization is gaining increasing traction among the stroke research community. The highest priority was given to research questions focused on life after stroke.

We can speculate on reasons for the shift from pathology-driven research questions to those valuing physical and psychological recovery. This could reflect the changing demographics of stroke care. Advances in acute stroke care have substantially improved stroke survival^2,24–27^ with a resulting increase in the number of people living with the consequences of stroke.^2,27^ It could be argued that the prioritization of postacute topics is a fair reflection of the state of stroke research. We have a firm evidence base for many acute stroke interventions, but the evidence underpinning longer-term treatments is less robust.

Our results suggest that stroke priorities are context-specific and ensuring representation from a range of stakeholders is key. Despite the projections for significant rises in stroke disease among low- and middle-income countries, no priority setting exercise had a focus on these countries. Our results suggest that we cannot assume that priorities from one healthcare context will be transferable to another. This inequality in the international understanding of research priorities should be tackled in future exercises.

There was greater inclusivity of stroke survivors in priority setting exercises over time. This is likely to reflect the broader recognition of the value of involvement of patients and the public in research. However, there is still work to be done as stroke survivors and their caregivers remain under-represented (~40%). Even where stroke survivors are included, there is still scope to improve representation. Of the few studies which provided demographics on participants, those included were not representative of the majority of stroke survivors and tended to have milder stroke deficits.

We identified that both physical and psychological recoveries after stroke are key priorities for stroke survivors. This is in keeping with a recent systematic review of qualitative studies identifying that psychoemotional support and physical recovery are key unmet needs for stroke survivors.^28^ Similarly, a recent policy analysis using patient and health professional interviews identified that significant unmet needs in rehabilitation, support, and information/education remained at 6 months poststroke.^29^ The results of our synthesis need to be interpreted in context. While we were inclusive in our approach to priority setting, some of the individual exercises had a particular remit (e.g. life after stroke and aphasia). These more focused exercises were predominantly based in the postacute space. This may have weighted the results of this review toward rehabilitation and recovery priorities. This does not weaken the importance of our summary but highlights that the stroke community recognizes the need to raise the visibility of research in this area. The recent UK-based, JLA exercise distinguished prevention, prehospital and acute care from rehabilitation, and long-term care.

Interestingly, while the stroke research landscape appears to have been influenced by these changing priorities, other chronic disorders such as dementia remain focused on delivering pathology-based research.^30^ This is likely to be due to the disparity in advances between the two diseases in this area, but may also reflect cultural differences in the research agendas of these diseases. In priorities among people with cancer, diagnosis, support, and needs of caregivers were highlighted as top priorities.^31^ The greater focus on rehabilitation and recovery in stroke priority setting may reflect the greater risk of long-term disability after stroke. Similarly, prioritization studies for other chronic diseases, such as type 2 diabetes and hypertension, focus on prevention and cure through lifestyle modifications or pharmacological approaches.^32,33^ However, priority setting relating to Parkinson’s disease mirrors the findings of this review, prioritizing advances in physical and psychological recovery, although primarily through a pharmacological rather than rehabilitation approach.^34^ Again, these differing research priorities may reflect differences in the nature, treatments, and degree of reversibility of these different conditions.

Our assessment of method and reporting quality offers clear guidance on areas that could be improved in future priority-setting exercises. Defining the key stakeholders and ensuring inclusion is key. However, to allow a diverse group of stroke survivors to participate may require adaptations to the usual methods of questionnaires and in-person group meetings. The major limitation seen in both the methods and reporting of priority setting was around the processes that followed creation of the priority list. Priority setting is only of value if people read and use the resulting priorities, yet few articles had plans for knowledge transfer, implementation, or evaluation of impact. Excepting the recent UK Stroke Association^8^ study, no studies in this review outlined a strategy to implement priorities into the research funding agenda.

Our review provides a collated set of stroke research priorities across a range of settings, times, and stakeholders. While we tried to be inclusive in our searching, it is possible that we missed non-English language articles that were not indexed on the databases we searched. To allow us to assess the methods and reporting of priority setting, we limited to articles with a description of the prioritization process. Thus, we did not include priorities only described in other formats, for example, on websites of third sector (voluntary, community, or charitable) organizations. Four included studies were not part of the original database search output. This reflects the diversity of publication and dissemination routes outside of academic journals for priority setting exercises, and also points to a need for standardization around the indexing of published priority setting research. We may have missed research prioritization exercises that were not stroke specific but included stroke content. For example, physical recovery or rehabilitation research may be more likely to be conducted by physiotherapists. There was insufficient detail in the published reports to assign priorities to specific stakeholder groups and future exercises may wish to include this level of detail.

### Implications for future research

Based on the findings from this review, we recommend future research focuses on those key areas that were consistently rated as high priority, particularly research around the life after stroke. While our approach does not allow for a short list of consensus priority research questions, it offers a useful synthesis to guide research. It is important to note that these priorities predominantly reflect those of high-income countries, and stroke survivors were under-represented. A set of internationally agreed research priorities would still have enormous value, particularly if the exercises give voice to low-middle-income countries and people with lived experience. Finally, our approach does not allow us to describe the effect of the prioritization on research funding or policy. Describing changes to the stroke research ecosystem before and after publication of the priority setting would be a useful next stage of research.

## Conclusion

Many stroke research priority setting exercises have been completed. These provide common messages around the need to promote research on life after stroke. Priorities are dynamic and context-specific. Few exercises have been conducted in low-middle-income countries. To ensure that relevant priorities are informing the research agenda, there is a need to regularly update the process and improve the inclusion of all relevant stakeholders, with a broader geographical scope.

## Supplemental Material

sj-docx-1-wso-10.1177_17474930221096935 – Supplemental material for International research priority setting exercises in stroke: A systematic review Supplemental material, sj-docx-1-wso-10.1177_17474930221096935 for International research priority setting exercises in stroke: A systematic review by Stephanie Leitch, Monica Logan, Lucy Beishon and Terence J Quinn in International Journal of Stroke
