# Supplementary material for: International research priority setting exercises in stroke: A systematic review
Source: Int J Stroke. 2022 May 12;18(2):133–43. doi: 10.1177/17474930221096935 (PMC13020986; doi:10.1177/17474930221096935)
Supplement: sj-docx-1-wso-10.1177_17474930221096935 – Supplemental material for International research priority setting exercises in stroke: A systematic review [file sj-docx-1-wso-10.1177_17474930221096935.docx]

**Supplementary material**

**Refined search syntax**

{Health Priorities/ and research*} {health research priorities AND stroke} {Research priority setting AND stroke} {Setting research priorities AND stroke}.

| **Category** | **Definition** | **Example Research Question** |
| --- | --- | --- |
|  |  |  |
| Prevention | Primary/secondary stroke prevention trials including medications and lifestyle factors. | Establish a global chronic disease prevention initiative that includes stroke as a major focus among a cluster of conditions including cardiovascular disease, diabetes mellitus, and dementia |
| Pathology | Mechanism of stroke, stroke subtypes, pre-clinical and translational stroke research. | Understand the cellular and molecular mechanisms with which endothelial cells of the BBB react to focal cerebral ischemia, identify targets to protect these cells, the BBB, and the neurovascular unit against damage. |
| Drug Interventions | Relating to hospital interventions and drug therapies. | Identify and test non-neurotoxic thrombolytics, understand mechanisms by which current thrombolytics damage vessels and neurons. |
| Physical Recovery | Trials or observational studies describing recovery from physical impairment or activity limitation following stroke. | The role of new technologies in improving physical recovery after stroke. |
| Psychological Recovery | Trials or observational studies describing recovery of cognitive, mood, psychiatric issues after stroke. | What are the best ways to manage and treat psychological impairment, cognitive impairment and fatigue after TIA/minor stroke? |
| Caregivers and Support groups | Impact of stroke on family members, support groups and health care professionals. | Health professionals (including local doctor), the stroke survivor, and their family or carers should all be involved in developing a plan. This plan is about stroke care after hospital. |
| Awareness | Education programmes aimed at the public or healthcare professionals surrounding stroke awareness/recognition. | Health professionals and the public should get education about how to recognize stroke early. That education needs to make it clear that stroke is a medical emergency. |
| Return to everyday life | Resumption of every day routine tasks, reintegration to the community and work (research at the level of societal participation). | What advice should healthcare professions give to TIA/minor stroke patients on return to work and activities? |
| Rehabilitation and Follow-up | General rehabilitation of stroke (not specified or limited to physical or psychological domains) or relating to longer-term (>1 year) follow up. | What is the most effective follow-up pathway for TIA/ minor stroke patients? |
| Other | Not related to any of the other defined categories. | Does fast-tracking potential stroke patients to comprehensive stroke units improve outcomes? |

*Supplementary Table 1. Thematic categories with associated definitions and one example priority research question.*

|  |  | Turner (2018) | Bayley (2003) | Alexandrov (2008) | Sacco (2015) | Sangvatanakul (2010) | Lannin (2012) | Meairs (2006) | Rowat (2009) | Franklin (2017) | Rowat Pollock (2016) | Pollock (2012) | National Stroke Foundation (2015) | Rudberg (2021) | James Lind Alliance (2021) |
| --- | --- | --- | --- | --- | --- | --- | --- | --- | --- | --- | --- | --- | --- | --- | --- |
| **Context and scope** | | | | | | | | | | | | | | | |
| 1 | Define geographical scope |  |  |  |  |  |  |  |  |  |  |  |  |  |  |
| 2 | Define health area, field, focus |  |  |  |  |  |  |  |  |  |  |  |  |  |  |
| 3 | Define end-users of research |  |  |  |  |  |  |  |  |  |  |  |  |  |  |
| 4 | Define the target audience of the priorities |  |  |  |  |  |  |  |  |  |  |  |  |  |  |
| 5 | Identify the broad research area |  |  |  |  |  |  |  |  |  |  |  |  |  |  |
| 6 | Identify the type of research question |  |  |  |  |  |  |  |  |  |  |  |  |  |  |
| 7 | Define the time frame |  |  |  |  |  |  |  |  |  |  |  |  |  |  |
| **Governance and team** | | | | | | | | | | | | | | | |
| 8 | Describe selection of the leadership and management team |  |  |  |  |  |  |  |  |  |  |  |  |  |  |
| 9 | Describe the characteristics of the team, and the networks they represent |  |  |  |  |  |  |  |  |  |  |  |  |  |  |
| 10 | Describe any training or experience in priority setting |  |  |  |  |  |  |  |  |  |  |  |  |  |  |
| **Framework for priority setting** | | | | | | | | | | | | | | | |
| 11 | State the framework used (if any) |  |  |  |  |  |  |  |  |  |  |  |  |  |  |
| **Stakeholders or participants** | | | | | | | | | | | | | | | |
| 12 | Define the inclusion criteria for stakeholders involved in priority-setting |  |  |  |  |  |  |  |  |  |  |  |  |  |  |
| 13 | State the strategy or method for identifying and engaging stakeholders |  |  |  |  |  |  |  |  |  |  |  |  |  |  |
| 14 | Indicate the number of participants and/or organisations involved |  |  |  |  |  |  |  |  |  |  |  |  |  |  |
| 15 | Describe the characteristics of stakeholders |  |  |  |  |  |  |  |  |  |  |  |  |  |  |
| 16 | State if reimbursement for participation was provided |  |  |  |  |  |  |  |  |  |  |  |  |  |  |
| **Identification and collection of research priorities** | | | | | | | | | | | | | | | |
| 17 | Describe methods for collecting priorities from stakeholders |  |  |  |  |  |  |  |  |  |  |  |  |  |  |
| 18 | Describe methods for collating and categorizing priorities |  |  |  |  |  |  |  |  |  |  |  |  |  |  |
| 19 | Describe methods and reasons for removing priorities |  |  |  |  |  |  |  |  |  |  |  |  |  |  |
| 20 | Describe methods for refining or translating priorities into research topics or questions |  |  |  |  |  |  |  |  |  |  |  |  |  |  |
| 21 | Describe methods for checking whether research questions or topics have been answered |  |  |  |  |  |  |  |  |  |  |  |  |  |  |
| 22 | Describe number of research questions or topics |  |  |  |  |  |  |  |  |  |  |  |  |  |  |
| **Prioritisation of research topics/questions** | | | | | | | | | | | | | | | |
| 23 | Describe methods and criteria for prioritising research topics or questions |  |  |  |  |  |  |  |  |  |  |  |  |  |  |
| 24 | Provide reasons for excluding research topics/questions |  |  |  |  |  |  |  |  |  |  |  |  |  |  |
| **Output** | | | | | | | | | | | | | | | |
| 25 | Specificity of research priorities are clear |  |  |  |  |  |  |  |  |  |  |  |  |  |  |
| **Evaluation and feedback** | | | | | | | | | | | | | | | |
| 26 | Describe how the process of prioritisation was evaluated |  |  |  |  |  |  |  |  |  |  |  |  |  |  |
| 27 | Describe the approach for feeding back priorities to stakeholders and/or to the public; and how feedback was addressed and integrated |  |  |  |  |  |  |  |  |  |  |  |  |  |  |
| **Implementation** | | | | | | | | | | | | | | | |
| 28 | Outline the strategy or action plans for implementing priorities |  |  |  |  |  |  |  |  |  |  |  |  |  |  |
| 29 | Describe evaluation of impact |  |  |  |  |  |  |  |  |  |  |  |  |  |  |
| **Funding and conflict of interest** | | | | | | | | | | | | | | | |
| 30 | State sources of funding |  |  |  |  |  |  |  |  |  |  |  |  |  |  |
| 31 | Outline the budget and/or cost |  |  |  |  |  |  |  |  |  |  |  |  |  |  |
| 32 | Provide declaration of conflict of interest |  |  |  |  |  |  |  |  |  |  |  |  |  |  |

*Supplementary Table 2. REporting guideline for PRIority SEtting of health research (REPRISE) checklist*
